# Fabricating customized hydrogel contact lens

**DOI:** 10.1038/srep34905

**Published:** 2016-10-17

**Authors:** Andre Childs, Hao Li, Daniella M. Lewittes, Biqin Dong, Wenzhong Liu, Xiao Shu, Cheng Sun, Hao F. Zhang

**Affiliations:** 1 Department of Physics and Astronomy, University of Texas at San Antonio, San Antonio, TX, 78249, USA; 2 Department of Biomedical Engineering, Northwestern University, Evanston IL 60208, USA; 3 Department of Mechanical Engineering, Northwestern University, Evanston IL 60208, USA; 4 Department of Ophthalmology, Northwestern University, Chicago IL 60611, USA.

## Abstract

Contact lenses are increasingly used in laboratories for *in vivo* animal retinal imaging and pre-clinical studies. The lens shapes often need modification to optimally fit corneas of individual test subjects. However, the choices from commercially available contact lenses are rather limited. Here, we report a flexible method to fabricate customized hydrogel contact lenses. We showed that the fabricated hydrogel is highly transparent, with refractive indices ranging from 1.42 to 1.45 in the spectra range from 400 nm to 800 nm. The Young’s modulus (1.47 MPa) and hydrophobicity (with a sessile drop contact angle of 40.5°) have also been characterized experimentally. Retinal imaging using optical coherence tomography in rats wearing our customized contact lenses has the quality comparable to the control case without the contact lens. Our method could significantly reduce the cost and the lead time for fabricating soft contact lenses with customized shapes, and benefit the laboratorial-used contact lenses in pre-clinical studies.

While contact lenses are commonly used as consumer medical devices for vision correction, they have been increasingly applied in laboratories for *in vivo* animal retinal imaging and pre-clinical studies^1^,^2^,^3^,^4^,^5^,^6^,^7^. Retinal diseases affect millions of people worldwide, resulting in loss of vision and diminished quality of life. In the path to fully understand the disease development mechanisms and to find the best treatment, *in vivo* retinal imaging of animal models has gained its popularity in studying retinal diseases^8^,^9^,^10^. It provides a cost-efficient solution to comprehensively investigate retinal disease pathophysiology and therapeutic effects of possible treatments. Custom-shaped contact lenses have been used on animal eyes to (1) minimize optical aberrations and (2) prevent corneal dehydration^11^,^12^. Eyeball optical aberration in species such as rats and mice is about five times higher compared with human eyes, which is one of the major factors limiting the resolution of animal retinal image^11^. A plano-concave contact lens can be used to reduce the geometrical aberration caused by cornea and thus improve image resolution in optical coherence tomography (OCT), fundus photography, two-photon retinal imaging, and confocal retinal imaging^1^,^2^,^3^,^4^,^5^. On the other hand, corneal dehydration is a potential side effect during animal retinal imaging when animals are under anesthesia and stop blink reflex. Corneal dehydration could cause corneal clouding which affects the imaging quality and even cause permanent corneal damage^13^. Thus, the contact lens covering the cornea will help to keep it moist to ensure consistent imaging results from various animal studies^14^,^15^. Those applications, however, often require modifying the shape of the contact lenses in order to achieve optimal fitting to the cornea of each individual test subject, but the choices from commercially available contact lenses are rather limited. Thus, it calls for the need for a flexible fabrication method to customize contact lenses that can be conveniently implemented in research laboratories.

While one would expect contact lenses to exhibit excellent optical transparency to accomplish the intended optical functions, they also need to be biologically compatible to maintain the corneal and general eyeball physiological conditions^16^. Currently, most contact lenses used in research imaging applications were made of poly(methyl methacrylate) (PMMA)^1^,^3^,^5^,^15^,^17^. PMMA is rigid and has poor oxygen permeability. Although PMMA contact lens preserves the water content of anterior ocular media for a short time, the limited oxygen permeability can cause corneal hypoxia, corneal edema, and corneal transparency deterioration in extended wearing, which is not desirable for *in vivo* studies^16^,^17^.

Hydrogel is found to be a more suitable material to construct contact lens for *in vivo* studies. Hydrogel contains 24% to 78% water in volume^18^,^19^. It is soft and highly permeable to oxygen compared with PMMA^17^. Hydrogel contact lens can greatly improve wearing comfort, and potentially prevent physiological changes in cornea while providing a good optical transparency during retinal imaging. Although not frequently used in laboratorial retinal imaging, hydrogel has gained its success in the commercial contact lens market as the material of soft contact lens^17^.

Despite the advantages, commercial hydrogel contact lenses for laboratorial use are rarely seen, due to the prohibitive cost for customization from leading manufacturers. Laboratory-use contact lenses often require unique shapes to fit both specific animal corneal curvature and imaging system, e.g., plano-concave contact lenses were used in confocal retinal imaging^2^,^3^. It is difficult to mass produce commercial hydrogel contact lenses to fulfill those requirements, due to the high cost for customization and small quantity in demand. Currently, only few rigid polymer (PMMA) animal contact lenses are in the market and no customizable hydrogel contact lens is commercially available^20^.

Here, we developed a lab-friendly method for fabricating customized hydrogel contact lenses, which is simple, rapid, and highly adjustable for particular needs in animal retinal imaging. We performed a comprehensive study to characterize material’s optical properties, including refractive indices and optical transparency, as well as its mechanical properties, including Young’s modulus and contact angle. We further tested the biological compatibility of our customized hydrogel contact lenses using live rats and quantified the imaging quality through the contact lens via *in vivo* OCT retinal imaging.

## Results

### Hydrogel synthesis and molding

We chose well-studied hydrogel as the material for soft contact lens to minimize the uncertainty in the imaging experiments. The procedure for hydrogel synthesis is adopted and simplified from previous reports to better fit the laboratorial uses^21^,^22^. We first combined 30 mL of 2-hydroxyethyl methacrylate (HEMA, containing monomethyl ether hydroquinone as inhibitor, Sigma-Aldrich) with 10 mg of inhibitor remover beads (inhibitor-removal 306312, Sigma-Aldrich) and stirred for 3 min to remove inhibitors. After filtering out the beads, we added 0.52 g of ethylene glycol dimethacrylate (EGDMA, Sigma-Aldrich) as crosslinking agent, 18.1 mL of deionized water, and 0.52 g of ultraviolet (UV) initiator (Irgacure 1173, BASF, OctoChem, Vandalia, IL) to HEMA. The mixture was then stirred until clear. The total solution by weight consisted of 63% HEMA, 1% EGDMA, 35% deionized water, and 1% UV initiator. The solution was then poured to different molds and polymerized by UV exposure (100 W, 365 nm center wavelength, 10 min exposure time) in a homemade UV chamber with inner walls and bottom covered by aluminum foil.

**Figure 1:**
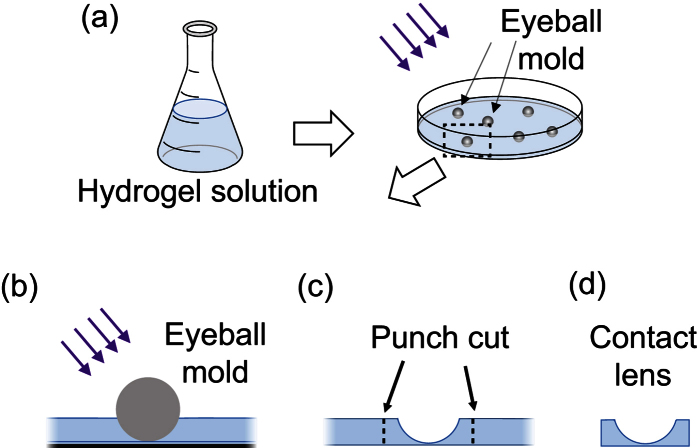
Schematic of fabrication of hydrogel contact lens using petri dish and steel balls as eyeball molds. (**a,b**) Hydrogel solution was added into a petri dish with a pre-polymerized thin hydrogel layer on bottom. Steel balls were placed in the petri dish as rat eyeball mold. Panel (b) is the magnified view of one eyeball mold highlighted by black dashed box in (**a**). Hydrogel was polymerized. The petri dish and the steel balls were then removed; Indented sections of hydrogel were punch cut (**c**) to form round-shape, plano-concave hydrogel contact lenses (**d**).

We fabricated HEMA hydrogel in the forms of thin slabs, cylinders, and plano-concave contact lenses for optical/mechanical property characterizations and retinal imaging tests, respectively. The thin slabs were fabricated by photopolymerizing the HEMA hydrogel solution sandwiched between two glass slides. The slab thickness was controlled by using spacers between the glass slides. Hydrogel cylinders were fabricated by polymerizing the solution in cylindrical molds, such as glass beakers. The fabrication of plano-concave contact lenses are shown in Fig. 1. We first polymerized a 200-μm-thick layer of hydrogel in a glass petri dish, and then added more hydrogel solution (about 2 mm in depth) on the top. In this study, the steel balls with 6 mm in diameter was used as the mold to match the shape of the rat cornea. After placing the steel balls in hydrogel solution, we polymerized the second layer. At the end, we peeled the molded hydrogel from the petri dish, and removed the steel balls. The indented sections of hydrogel were cut out for *in vivo* rat retinal imaging tests.

### Optical refractive index and transmission spectra

**Figure 2:**
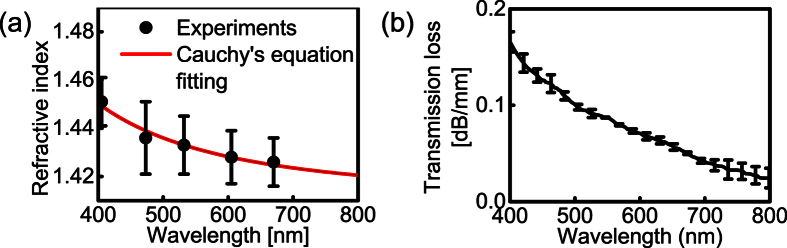
Optical properties of the synthesized hydrogel. (**a**) Refractive indices (measured experimentally under the wavelengths of 405 nm, 473 nm, 532 nm, 605 nm and 670 nm) and the fitted dispersion curve. (**b**) The transmission loss within the visible light range.

Optical refractive index is essential to lens materials. A high refractive index could reduce lens thickness and improve wearing comfort^23^. We determined the refractive index of our hydrogel by measuring the light refraction in the material. A collimated laser beam (1 mm in beam diameter) illuminated from air into the side of a bulk hydrogel cylinder, and the optical path was recorded from the top of the cylinder. We measured the angles of incidence and refraction and calculated hydrogel’s refractive index using Snell’s law: *n* = sin *θ*_in_/sin *θ*_re_, where *n* is the refractive index of the hydrogel; *θ*_in_ is the angle of incidence; and *θ*_re_ is the angle of refraction. We used five laser lights with different wavelengths (405 nm, 473 nm, 532 nm, 605 nm, and 670 nm) to sample the visible spectral range. The angle of refraction was measured six times for each wavelength to calculate the mean value. We then fit the refractive index dispersion curve in the wavelength range of 400 nm to 800 nm using Cauchy’s equation: *n*(*λ*) = *B* + *C*/*λ*^2^, where *B* and *C* are empirical coefficients that can be determined through curve fitting^24^. As shown in Fig. 2a, the refractive index of our synthesized hydrogel was 1.42 to 1.45 (3% measurement variation) in the wavelength range from 400 nm to 800 nm, which is comparable to the value of 1.40 to 1.43 of commercial soft contact lenses^23^.

The transparency is a fundamental requirement for contact lens materials. We measured the transmission spectrum of our fabricated hydrogel using a commercial microscope (DMI3000M, Leica) equipped with a spectrometer (Shamrock 303i, Andor Technology. 150-lines per mm of grating density, 0.88 nm of spectral resolution). White illumination light was weakly focused on the hydrogel thin slabs. The transmitted light was collected by the objective lens and relayed to the spectrometer to measure the transmission spectra.

The optical extinction is collectively determined by (1) reflection and scattering from the hydrogel surface, (2) hydrogel’s intrinsic absorption loss, and (3) volumetric scattering loss. The latter two determine the actual attenuation caused by the hydrogel material. We first estimated the optical loss from the hydrogel surface. Since the roughness of the hydrogel surface is determined by the glass slides used in fabrication, which are normally optically smooth (less than 2 nm in surface roughness)^25^, we assumed the light scattering from surfaces of hydrogel slabs is negligible, and the surface optical loss is mainly from reflection. Thus, to determine the actual optical attenuation induced by the hydrogel, we estimated the reflection from two air-hydrogel interfaces using our experimentally measured refractive index in Fig. 2a, and then subtracted this reflection loss from the transmission spectrum shown in Fig. 2b. Figure 2b shows high optical transmission within the spectral range from 400 nm to 800 nm. When the slab thickness is comparable to that of commercial contact lenses, e.g. 0.5 mm, the average loss caused by our hydrogel material is less than 2% within 400 nm to 800 nm wavelength range, which is minor compared with the surface reflection losses.

### Young’s modulus

Young’s modulus is critical for contact lens wearing comfort and vision correction accuracy^26^. Low modulus could increase wearing comfort, while the material also needs to be sufficiently stiff to maintain the shape during wearing. Thus, the modulus of commercial contact lenses is commonly found within the range from 0.3 to 1.9 MPa^26^.

**Figure 3:**
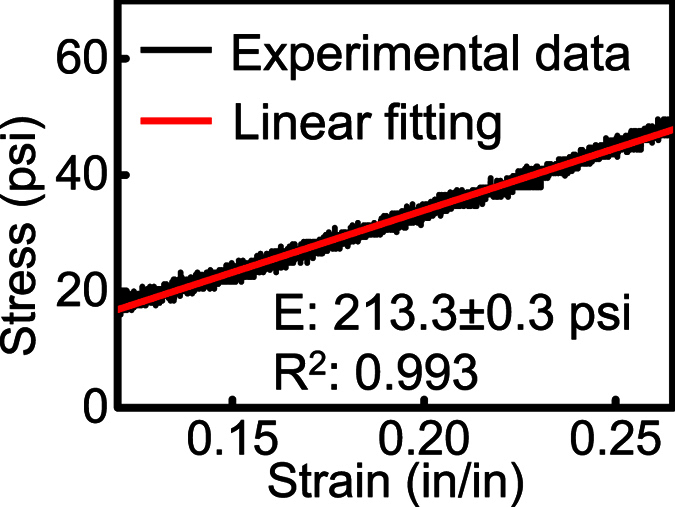
Stress vs. strain from the cylinder compression test. Black dots: experimental data. Red line: linear fitting. E: Young’s modulus. R^2^: adjusted coefficient of determination.

We measured the hydrogel’s Young’s modulus by standard cylinder compression test. A hydrogel cylinder (25 mm in height, 25 mm in diameter) was tested by a servo hydraulic system (model 8500, Instron). The measured deformations under different compressional loadings were used to calculate the Young’s modulus. The result of the stress vs. strain is shown in Fig. 3. The Young’s modulus, calculated from the stress-strain ratio, is 1.47 MPa (213.3 psi), which is within the range of commercial soft contact lens materials (larger than senofilcon A and etafilcon A, comparable to balafilcon A, and smaller than lotrafilcon A)^26^. Noticeably, the test is on a bulk material. Contact lenses are normally thinner than 1 mm and may have a reduced Young’s modulus^27^. It may explain why our value is relatively larger than the reported values of other hydrogel materials with the similar water content, which are normally obtained from thin slabs.

### Sessile drop contact angle

Contact angel characterizes material’s hydrophobicity (or wettability). It shows how easy water can spread on a solid surface. For contact lenses, hydrophilic surfaces with small contact angles are desired to support a ocular tear film between the lens and the cornea, and to increase the wearing safety and comfort^28^. Contact angles for commercial soft contact lenses range from 15° to 80°, depending on different materials^28^.

**Figure 4:**
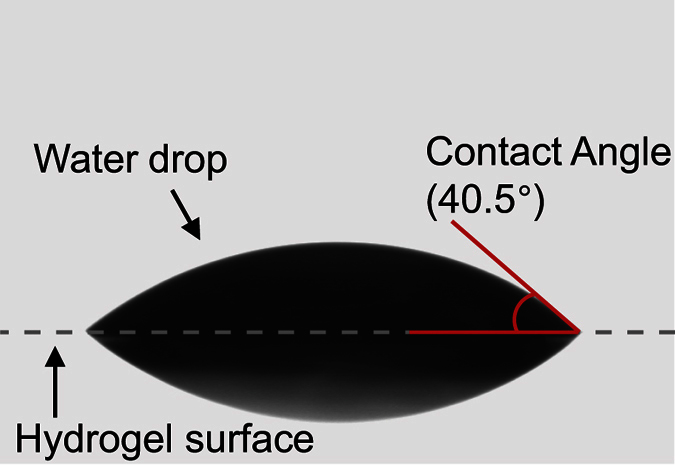
Hydrophobicity test using the static sessile drop measurement.

We used a standard static sessile drop method (VCA Optima XE, AST Products) to characterize hydrogel’s hydrophobicity. We placed a 2-μL water droplet on the surface of a hydrogel thin slab and took a side-view photo to measure the contact angles. We repeated the measurement three times and calculated the mean value. The contact angle of our hydrogel is measured to be 40.5° ± 0.5°. A representative image of sessile drop measurement is shown in Fig. 4. Our contact angle is smaller than that of senofilcon A and balafilcon A, comparable to lotrafilcon A, and larger than etafilcon A, showing a hydrophobicity comparable to commercial hydrogel materials^28^.

### OCT imaging

We used a home-built near-infrared OCT system (center wavelength 840 nm, bandwidth 95 nm) to image the rat retina through a fabricated hydrogel contact lens, and compared the results with the image obtained without the contact lens and with a commercial PMMA contact lens (Metro Optics, 6 mm diameter, zero optical diopter). Details of the OCT system was described previously^29^. When imaging the retina without using the contact lens or using the PMMA lens, we used a relay-lens group to focus the light onto the retina. When imaging the rat retina through the hydrogel contact lens, as the result of the plano-concave contact lens, most dioptric power of the rat eyeball was cancelled and a scanning lens can be directly used to image the retina. To achieve a fair comparison, we set the same numerical apertures for both the lens-relay system and the scanning lens system to be 0.04, giving a lateral resolution of 12 μm. The axial resolutions are 3 μm for both systems, determined by the center wavelength and bandwidth of the OCT light source. With the similar imaging power, the scanning lens system could inspect the opacity of the contact lens material and eyeball condition changes (e.g., corneal clouding) by comparing the imaging qualities from two systems. During imaging, we acquired 256 × 256 A-lines within a 1.2 mm × 1.2 mm retinal area. The acquisition time was about 2 seconds per image.

**Figure 5:**
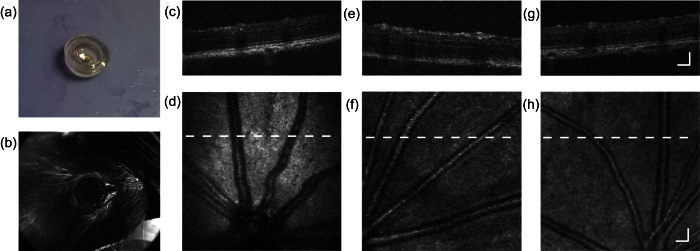
*In vivo* OCT imaging of rat retinae with and without the contact lens. (**a**) The hydrogel contact lens designed for rat eye. (**b**) A picture of rat eye wearing the contact lens. (**c**) One B-scan of rat retina with the hydrogel contact lens. (**d**) En face retinal image through the hydrogel contact lens. (**e**) One B-scan of rat retina with a commercial PMMA contact lens. (**f**) En face retinal image through the PMMA contact lens. (**g**) A B-scan of rat retina without contact lens. (**h**) En face retinal image without contact lens. White dash lines in panels (d,f,h) are the locations of the B-scan images in panels (c,e,g), respectively. Bar: 100 μm.

We used wild-type pigmented rats for OCT imaging. Pictures of a hydrogel contact lens and a rat wearing the hydrogel contact lens are shown in Fig. 5a,b, respectively. The lens fitted the rat eyeball well and covered the whole cornea. No bubble was observed between the cornea and contact lens. The retinal images from the rat eyes with and without the contact lenses are shown in Fig. 5c to Fig. 5h. Typical OCT B-scans from eyes without the contact lens, with PMMA lens and with hydrogel lens are shown in Fig. 5c,e,g, respectively. All images clearly show retinal vessel cross sections and different layers of the retina, indicating that the contact lens did not deteriorate the axial resolution of the OCT. The *en face* OCT images shown in Fig. 5d,f,h are the mean-value-projections from each A-line. Main vessels are clearly shown in all images, and some retinal arterioles and venules can also be seen but not clearly resolved, which is due to the limited contrast of blood within the NIR spectral range.

We used the quality index (*QI*) to quantitatively compare the OCT image qualities^30^. *QI* evaluates the OCT image quality from reflectivity values in each B-scan, which is more direct and accurate than analyzing from *en face* images, since *en face* images are derived from B-scans and some information might be lost during mean-value projection. *QI* is defined as


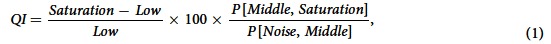


where *Low*, *Noise*, and *Saturation* are the pixel intensities at the first percentile, 75th percentile and 99th percentile of all recorded pixel intensities in the image histogram, respectively. *Middle* is the mean value of *Noise* and *Saturation. P* [*Middle, Saturation*] is the number of pixels between *Noise* and *Saturation* intensity values and *P* [*Noise, Middle*] is the number of pixels between *Noise* and *Middle* Intensity values. In the previous report, images with a *QI* score of 34.6 or higher was considered “excellent”^30^. We calculated the averaged *QIs* from B-scans of Fig. 5d,f,h, and the scores are 92 ± 5, 116 ± 8 and 111 ± 6, respectively. All scores are more than double of the criterion of “excellent”, showing that high-quality OCT retinal images could be obtained through our hydrogel contact lens.

The highest *QI* is from retinal images through PMMA contact lens, which is consistent with the previous report^1^. The *QI* from the retinal image through hydrogel contact lens is slightly lower than that without contact lens and with PMMA contact lens. It might be due to the image off-focus in the peripheral retinal area when using a scanning lens. When imaging a retina without contact lens, a collimated light beam illuminated on the cornea, and was well focused on the retina by cornea and ocular lens in a large field of view. When using a focused beam to image a retina through a plano-concave contact lens, the light was focused on a flat focal plane. Such imaging setup could cancel the optical aberration from the cornea and increase the imaging resolution. However, only the center retinal area could be kept in focus as the retina is curved and the peripheral area will be blurred, which could contribute to their relatively lower *QI* score. Such image blur in the peripheral area is also observed in Fig. 5c,d. We cropped the peripheral area in Fig. 5d (cutting 30% from each side) and the *QI* is calculated to be 113 ± 5, which is comparable to that of wearing the PMMA contact lens.

We demonstrated that high-quality OCT images can be acquired with our hydrogel contact lens in the near infrared range (792 nm to 888 nm). Considering the low transmission loss in visible spectral range, we believe our hydrogel contact lens is also compatible with retinal imaging modalities using visible light, such as fundus photography, visible-light confocal retinal imaging and visible-light OCT^2^,^4^,^5^,^31^.

### The Influence of the hydrogel contact lens on rat eyeballs

We evaluated the potential influence of the contact lens on the rat cornea by quantitatively examining its influence on fundus images over the duration of one hour. We placed the contact lens only on the left eye while using the right eye as a control. For the left eye, we removed the contact lens every 30 min and acquired a fundus image, and then placed the contact lens back. For the right eye, we also took fundus images every 30 min for comparison. We applied artificial tears on the right eye to keep the cornea moist between each inspection. The quality of fundus images was then evaluated for any possible corneal clouding. We acquired the fundus images using a home-made high-resolution fundus camera^32^. The exposure time of fundus imaging was 0.2 s; the image resolution was 10 μm; and the field of view was 50 degrees. We also performed visual check of the corneal clouding on the second day and the sixth day after the initial placement of the contact lens.

**Figure 6:**
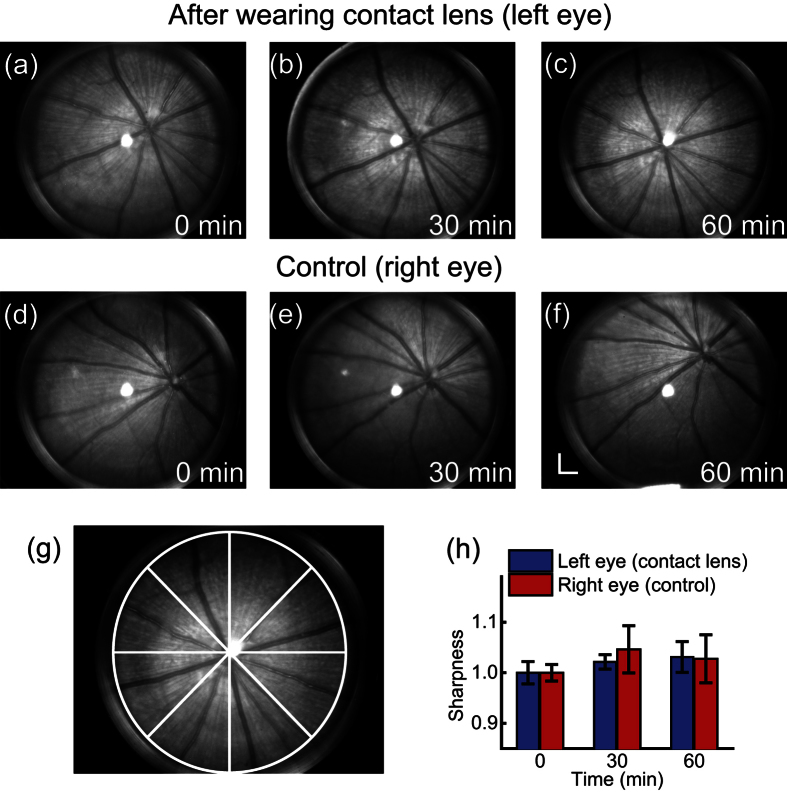
Fundus images of rat eyes with and without the hydrogel contact lens. (**a**) to (**c**) Fundus images of the left eye before wearing contact lens, 30 min and 60 min after wearing contact lens, respectively. (**d**) to (**f**) Images of the right eye without wearing contact lens. Bar: 500 μm. (**g**) A retinal image divided into 8 fan-shaped sections for sharpness analysis. (**h**) Sharpness variation in one-hour observation. The sharpness values at 30th minute and 60th minute are normalized to the value at 0 minute.

In the one-hour cornea inspection, we did not observe any clouding on either the contact lens-wearing eye or the control eye. The fundus images are shown in Fig. 6a to Fig. 6f. We compared the image qualities using a sharpness metrics analysis^32^,^33^. The sharpness *M* is defined as:


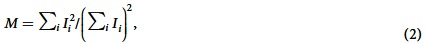


where *I*_*i*_ is the intensity of the *i*’th pixel, and the summation is taken in a selected region of interest. As shown in Fig. 6g, we divided one retinal image into eight fan-shaped regions to calculate the individual sharpness values, and then calculated the mean sharpness value and standard deviation from these eight regions. The sharpness variation in one hour is shown in Fig. 6h. There is no obvious sharpness deterioration, which is consistent with our visual inspection of cornea. We did not observe any obvious corneal clouding on the second day and the sixth day after this study. These observations suggest no obvious negative effect on rat eye cornea by wearing the contact lens.

One hour of wearing contact lens may be sufficient for most short-time laboratorial eye imaging acquisitions, but may not be enough for long-time retinal monitoring during animal eye surgery or in retinal disease studies^6^,^7^. In the future, evaluation of eyeball qualities with longer contact lens wearing time will be necessary.

## Discussion

We report in this paper a cost effective method for rapid fabrication of customized contact lenses. In comparison, commercial soft contact lenses are normally fabricated by using expensive and time consuming lathe cutting or injection molding methods^17^. The lathe cutting method needs high-end lathe machine and precise computer control. The materials also require to facilitate non-hydrated/hydrated phase changes from hard lathing target to soft material. Although highly automated in recent years, the whole process is still not easily accessible for research labortories^17^. On the other hand, the injection molding method has been optimized for mass production but it lacks the flexibility to rapid fabrication of customized contact lens in a cost effective manner^17^. In contrast, our fabrication method can be implemented in a laboratorial environment, using only common tools such as beakers, filter papers, and a UV lamp. The fabrication process reported in this work only takes about 20 min, which is obviously more efficient for lab users.

Our hydrogel fabrication method is compatible with both traditional hydrogel and silicone hydrogel contact lens. The material of our fabricated contact lens is polymerized HEMA, which is considered as a traditional hydrogel material. Silicone hydrogel is a type of silicone-monomer-doped hydrogel, which can further increase the oxygen permeability at the cost of decreased water content and wettability^17^,^34^. In principle, our method could also be applied for silicone hydrogel fabrication in laboratory since silicone hydrogel could also be polymerized by UV light. Previous report has demonstrated fabricating the traditional hydrogel and silicone hydrogel using almost the same procedures in laboratorial environment^22^. To fabricate customized silicone hydrogel contact lens, one could add the silicone monomers, e.g., mix methacryloxypropyltris (trimethylsiloxy) silane (TRIS), with HEMA solution and keep the molding fabrication steps the same.

One potential important application of hydrogel contact lenses is the sustained ophthalmic drug release^35^. Our hydrogel is similar to the material reported in the ref. 21 and 22, which have been applied in the slow release of hyaluronic acid^22^, antibiotic ciprofloxacin^21^ and timolol maleate (for glaucoma treatment)^36^, suggesting that our hydrogel should be applicable to drug loading and releasing studies. During fabrication, drug molecules can potentially be added to the solution before polymerization. Our fabrication process is performed under room temperature and pressure, which will help to preserve the drug activities. For particular drugs, high-power, short-wavelength UV exposure during UV curing might affect drug activities. It could be avoided by using longer wavelength and lower power at the cost of increasing exposure time.

The shape of our contact lens is currently coarsely controlled under a simplified molding process. The lens curvature is controlled by the steel beads as eyeball molds. Although different curvatures are achievable using beads with different diameters, the lenses are still bulky compared with thin, meniscus commercial contact lenses. It can be improved by using better molds without adding extra fabrication steps, e.g. molds used in industrial injection molding process, or customized molds using 3D printing. Noticeably, the hydrogel, as a photo-polymerized material, is possible for customized shaping using light-curing 3D printing^37^. The 3D-printed contact lens could provide more flexibility on customization, which may further benefit clinical needs of customized contact lenses, such as contact lenses for infants and patients after refractive surgeries^38^,^39^.

## Conclusion

We proposed a flexible method to fabricate customized hydrogel contact lenses which meets the various requirements in laboratorial research. We showed that the fabricated hydrogel has good optical and mechanical properties comparable to commercial-grade contact lens materials. The hydrogel is highly transparent, with a high refractive index in 400 nm to 800 nm wavelength range. The Young’s modulus and hydrophobicity are also comparable to commercial hydrogel materials. *In vivo* retinal imaging tests in rats wearing the hydrogel contact lens showed no obvious corneal damage and good compatibility with OCT imaging technology. This convenient and flexible method could potentially significantly reduce the cost of in-lab contact lens uses and help to unleash the potential of hydrogel contact lenses in various eye imaging and the pre-clinical studies in ophthalmology and ocular pharmacology. In the future, we plan to measure oxygen permeability, fabricate meniscus contact lenses, and test influence of long-time lens wearing.

## Methods

### Animal preparation

Wild-type pigmented rats were used in this study (250-g Long Evans rats, Charles River Laboratories). Rats were anesthetized by isoflurane mixed with normal air (2% isoflurane at 3 L/min for the first 10 min and 1.5% at 2 L/min in following experiments). Rat eyes were anesthetized using one drop of 0.5% Tetracaine Hydrochloride ophthalmic solution and dilated using one drop of 1% Tropicamide ophthalmic solution. During imaging, animals were restrained on a homemade holder. We added two drops of artificial tears in the bowl of hydrogel contact lens and gently placed the lens on the rat’s cornea. Special care was taken to squeeze out any air bubble between the contact lens and rat cornea. All experiments were performed in compliance with the ARVO Statement for the Use of Animals in Ophthalmic and Vision Research, and were approved by the Animal Care and Use Committee of Northwestern University.

## Additional Information

**How to cite this article**: Childs, A. *et al.* Fabricating customized hydrogel contact lens. *Sci. Rep.* **6**, 34905; doi: 10.1038/srep34905 (2016).
